# Regulation of MMP-9 by a WIN-Binding Site in the Monocyte-Macrophage System Independent from Cannabinoid Receptors

**DOI:** 10.1371/journal.pone.0048272

**Published:** 2012-11-06

**Authors:** Svantje Tauber, Katrin Paulsen, Susanne Wolf, Peggy Synwoldt, Andreas Pahl, Regine Schneider-Stock, Oliver Ullrich

**Affiliations:** 1 Institute of Anatomy, Faculty of Medicine, University of Zurich, Zurich, Switzerland; 2 Institute of Immunology, Otto-von-Guericke-University Magdeburg, Magdeburg, Germany; 3 Nycomed Germany Holding GmbH, Konstanz, Germany; 4 Institute of Pathology, Erlangen, Germany; 5 Institute of Pathology, Otto-von-Guericke-University Magdeburg, Magdeburg, Germany; 6 Department of Machine Design, Engineering Design and Product Development, Institute of Mechanical Engineering, Otto-von-Guericke-University Magdeburg, Magdeburg, Germany; 7 Zurich Center for Integrative Human Physiology (ZIHP), University of Zurich, Zurich, Switzerland; 8 Neuroscience Center Zurich, University of Zurich and ETH Zurich, Zurich, Switzerland; University Hospital Freiburg, Germany

## Abstract

The cannabinoid system is known to be involved in the regulation of inflammatory processes. Therefore, drugs targeting cannabinoid receptors are considered as candidates for anti-inflammatory and tissue protective therapy. We demonstrated that the prototypical cannabinoid agonist R(+)WIN55,212-2 (WIN) reduced the secretion of matrix metalloproteinase-9 (MMP-9) in a murine model of cigarette-smoke induced lung inflammation. In experiments using primary cells and cell lines of the monocyte-macrophage-system we found that binding of the cannabinoid-receptor agonist WIN to a stereo-selective, specific binding site in cells of the monocyte-macrophage-system induced a significant down-regulation of MMP-9 secretion and disturbance of intracellular processing, which subsequently down-regulated MMP-9 mRNA expression via a ERK1/2-phosphorylation-dependent pathway. Surprisingly, the anti-inflammatory effect was independent from classical cannabinoid receptors. Our experiments supposed an involvement of TRPV1, but other yet unidentified sites are also possible. We conclude that cannabinoid-induced control of MMP-9 in the monocyte-macrophage system via a cannabinoid-receptor independent pathway represents a general option for tissue protection during inflammation, such as during lung inflammation and other diseases associated with inflammatory tissue damage.

## Introduction

In the last years, several *in vitro*, *in vivo* and clinical studies suggested that the endocannabinoid system (ECS) is a crucial participant in the control and regulation of inflammation, where it interferes at different points and in key mechanisms of the orchestrated immunological network. Cannabinoids inhibit the release of proinflammatory cytokines such as TNF-α, IL-1-β [1], [2] IL-2 (2), IL-6 and IL-8 [3], [4], and they apparently stimulate nitric oxide release [5]. It has been proposed that endocannabinoids are chemo attractants, which first help to attract macrophages to the site of tissue damage [6]. Beyond inflammatory mediators [7], essential immunological functions such as migration [8], chemotaxis [9] and immune cell apoptosis [10] are affected by cannabinoid signaling. Numerous *in vitro* and *in vivo* studies suggest that drugs targeting cannabinoid receptors or modulating tissue levels of endocannabinoids represent promising candidates for treatment of inflammatory conditions [11], [12], [13].

Throughout the animal kingdom the endocannabinoid system is a highly conserved signaling system, and it is already developed in invertebrates [14] and plants. The fact that even plants possess a signal transduction system which exceedingly resembles the endocannabinoid system in animals, underlines the success of this evolutionary achievement [15]. Both cannabinoid receptor 1 (CB1) and cannabinoid receptor 2 (CB2) are seven-transmembrane G_i/o_ -protein-coupled receptors, but distinct in distribution and physiological function. CB1 receptors are one of the most abundant G-protein-coupled receptors in the brain and mostly expressed on neurons of the neocortex, hippocampus, basal ganglia, cerebellum and brainstem [16], where they also mediate most of the effects of Δ^9^-tetrahydrocannabinol (THC) [16], [17]. CB2 receptors mediate anti-inflammatory effects in cells of the immune system [7], [18]. However, several studies have shown that some effects of cannabinoid ligands cannot be attributed to CB1 or CB2 receptors and several sites distinct from CB receptors, where at least some cannabinoid receptor ligands show activity, have been identified [19]. Among these sites are the non-selective cation channel transient receptor potential vanilloid type 1 (TRPV1) [20], the G protein-coupled orphan receptor GPR55 [21], [22] and the family of peroxisome proliferator-activated receptors (PPARs) [23]. Today pharmacological modulations of the cannabinoid system offer the opportunity for therapeutic intervention and the possibility to control or limit inflammation and to reduce tissue damage [24], [25].

Severe tissue damage and destruction during inflammatory conditions are often induced by collagen degrading proteins of the matrix metalloproteinase (MMP) family. These proteins are involved in the breakdown of extracellular matrix during development, tissue remodeling and cell migration during physiological conditions. The family of MMPs comprises almost 30 members of zink-dependent endopeptidases. Together they are capable of digesting all components of the basal membrane and the extracellular matrix and they constitute a crucial element of immune regulation [26]. MMPs are secreted by macrophages and other types of migrating cells and their obvious function is to enable cells to overcome physical barriers and allow them to infiltrate tissue [27]. Furthermore, MMPs have important immunregulatory functions including modulation of cytokines, chemokines and leukocyte recruitment [26], [28]. MMPs are considered to be involved in numerous cell functions in health and disease [29], [30], [31]. Among all MMPs, MMP-9 is regarded as a higher-ranking immune-modulatory element [32] and its secretion is enhanced in response to inflammatory stimuli such as TNF-α [33]. In order to avoid destructive activity, MMP-9 is regulated tightly at different levels: MMP-9 is secreted in a zymogenic form (92 kDa) following proteolytic cleavage yielding the active form (85 kDa) [34]. Other control-mechanisms are transcriptional regulation [35], post-translational regulation, regulation of the secretory process [36], and regulation by inhibitors of matrix metalloproteinases (TIMPs) [37]. MMP-9 plays a physiological role in tissue reorganization and bone metabolism [38], [39], [40], where it represents one of the most abundant proteases in osteoclasts [39], [41]. MMP-9 is furthermore involved in the pathophysiological process underlying many inflammatory diseases: It was already shown in 1999 that genetically MMP-9-deficient mice develop more severe experimental autoimmune encephalomyelitis (EAE) than wildtype littermates [42]. In multiple sclerosis MMP-9 causes increased permeability of the blood brain barrier, leading to greater inflammatory infiltration and it subsequently enhances demyelinisation and neuronal damage [43]. Inhibition of MMP-9 is discussed as a therapeutic strategy for multiple sclerosis, since inhibition of MMP-9 by Interferon-β led to reduced transmigration and decreased permeability of the blood brain barrier in patients [43], [44], [45]. In cardiovascular diseases MMP-9 contributes to the development of artheriosclerotic plaques and the resulting risk of strokes and cardiac infarction [46]. Furthermore, MMP-9 is involved in the pathogenesis of inflammatory bowel disease, rheumatoid arthritis and chronic obstructive pulmonary disease (COPD) [43], [47], [48]. Thus, MMP-9 is a key mediator of tissue destruction in many diseases marked by inflammatory tissue damage, and inhibition of MMP-9 seems a promising strategy to treat inflammatory diseases.

Because cannabinoids are able to modulate secretion of MMPs in different cell types such as epithelial cells [49], fibroblasts [50] and glioma cells [51], we aimed to investigate if macrophageal secretion of MMP-9 can be modulated by cannabinoids. In our study we used the aminoalkylindole compound WIN (R(+)WIN55,212-2 = [(3R)-2,3-dihydro-5-methyl-3-(4-morpholinylmethyl)pyrrolo[1,2,3-de]-1,4-benzoxazin-6-yl]-1-naphthalenyl-methanone, monomethanesulfonate, CAS No 131543-23-2), a prototypical cannabinoid, which is an agonist of cannabinoid receptor 1 and 2 and has been widely used to study cannabinoid signalling *in vitro* as well as *in vivo*. Its *in vitro* effects comprise modulation of chemokines, migration and proliferation [52], [53], [54]. Importantly, during the last years a rising number of *in vivo* studies have shown therapeutic effects of WIN in animal-models of inflammatory pain, arteriosclerosis and multiple sclerosis [55], [56], [57], [58]. Interestingly, some of its beneficial effects seem to be independent of cannabinoid receptors 1 and 2 [59], [60].

We combined investigations in primary cells and cell lines of the monocyte-macrophage system. We also looked at an animal model for lung inflammation. In these we examined the regulation of MMP secretion by cannabinoids and its underlying cellular mechanism in order to evaluate the potential of cannabinoid compounds in the treatment of diseases that comprise MMP-9-mediated tissue-destruction.

## Materials and Methods

### Reagents and Supplements

If not stated differently, reagents were purchased from Sigma Aldrich (Buchs, Switzerland). S(–)-WIN 55,212-3 and (R)-(+)-WIN 55,212-2 were dissolved in Dimethyl sulfoxide (DMSO) (10 mM). Phorbol-12-myristat-13-acetat (PMA) was dissolved in DMSO (1 mg/ml), pertussis toxin (Calbiochem, Darmstadt, Germany) was received as glycerol solution of 0.5 µg/ml, AM630 and AM251 (Tocris Laboratories, Bristol, United Kingdom) were dissolved in DMSO (100 mM), LPS (lipopolysaccharide) from *E. coli* 0111:B4 (Sigma) was dissolved in water (1 mg/ml), 1,3-Dimethoxy-5-methyl-2-[(1*R*,6*R*)-3-methyl-6-(1-methylethenyl)-2-cyclohexen-1-yl] benzene (O-1918) (Tocris) was dissolved in DMSO (100 mM), and (*R*)-(+)-*trans*-4-(1-Aminoethyl)-N-(4-Pyridyl)cyclohexanecarboxamide dihydrochloride monohydrate (Y27632) (Calbiochem) was purchased as a 5 mM aqueous solution. Dexamethasone was dissolved in ethanol (EtOH) (1 mg/ml). Receptor Activator of NFkB Ligand (RANK-L) and human macrophage colony stimulating factor (M-CSF) (PeproTech, London, UK) were dissolved in demineralized water (50 µg/ml and 25 µg/ml), human TGF-β 1 (PeproTech) was dissolved in phosphate buffered saline (137 mM NaCl, 2.7 mM KCl, 100 mM Na2HPO4, 2 mM KH2PO4) (PBS) and 2 mg/ml bovine serum albumin (BSA) (5 µg/ml). Capsaicin and Capsazepine were dissolved in EtOH (1 mM and 50 mM). Prior to treatment the substances were further diluted if necessary. In the cell culture experiments with the mentioned substances, the solvent concentrations never exceeded 0.1%. Vehicle controls were performed using the same solvent concentrations as in the experiments with the treatment-substances.

### Mouse Model of Smoke-induced Lung Inflammation

Male mice (C57BL/6J) aged 8–10 weeks were used (with a weight of 20–25 g) and obtained from Charles River (Sulzfeld, Germany). All animal experimentation/studies were performed in accordance with the national animal protection rules and they were approved by the local governmental authority (Ministerium für Landwirtschaft, Umwelt und ländliche Räume des Landes Schleswig-Holstein) under the permission no. V 312-72241.123-15 (74-7/10). The mice were housed in groups of 8–10 in individually ventilated microisolator-cages (IVC). Room temperature was kept at 22°–24°C, and relative humidity at 40–50%. Food and water were supplied *ad libitum*. WIN55212-2 mesylate (Cay100009023-50) was purchased from Caymen Chemicals. All drugs prepared in DMSO were injected intraperitoneally (3% DMSO) in volumes of 200 µl/animal. The solvent/vehicle alone had no effect on the response in the *in vivo* studies. WIN55212-2 mesylate was given i.p. daily 1 hour prior to cigarette smoke (CS) exposure. Mice (*n* = 8−10/group) were exposed (whole body) to CS (Reference Cigarettes 2R4F with filter, University of Kentucky, Lexington, KY, USA) 2 times a day for 1 h with 1 h smoke-free intervals, for 4 days. Control groups were exposed to air. For exposure mice were separated in custom-made steal cages to assure the whole body exposure of each mouse. The smoke was produced by the burning of cigarettes in a smoke generator (15 puffs/min) and was introduced into the exposure chamber with the airflow generated by a mechanical ventilator at a rate of 1.5 l/min. The smoke stream was diluted with compressed air (40 l/min). The total particulate matter in the CS was max. 700 µg/l.

### Bronchoalveolar Lavage

One hour after the last CS exposure the mice were sacrificed by intraperitoneal injection of an overdose of thiopental (Trapanal ®). The trachea was exposed and cannulated with a tracheal catheter. Bronchoalveolar lavage (BAL) was performed by instilling 2 x 0.8 mL of phosphate buffered saline (1 x PBS; Invitrogen cat no18912-014) supplemented with 0.5% bovine serum albumin (BSA) (Serva, Heidelberg, Germany). Total and differential cell count was determined automatically using a XT-2000iV haemocytometer (Sysmex, Norderstedt, Germany). The gates for the differentiation of cell types were adjusted to reflect the microscopic analysis of cytospin slides according to standard morphological criteria. The BAL fluid was centrifuged (1400 rpm, 10 min, 4°C). Supernatants were stored at −20°C.

### Cell Culture of U937 Monocytic Cells

U937 monocytic cells (ATCC Manassas, VA, USA) were grown in RPMI 1640 medium (Biochrom, Berlin, Germany) supplemented with 10% fetal bovine serum (FBS) (Biochrom), penicillin (100 U/ml) and streptomycin (100 mg/ml) in a 5% CO_2_ atmosphere at 37°C. Cell density was 0.15 - 2 x 10^6^ cells/ml, medium was changed every third day. Differentiation into macrophagial cells was induced by treatment with 100 nM PMA for 72 h at a cell concentration of 0.4 x 10^6^ cell/ml in cell culture plates (Nunc, Wiesbaden, Germany). PMA was first diluted in DMSO at 100 mM and then added to the cells in a dilution of 1∶1000, so that the final concentration of DMSO in the culture medium was 0.1%, which elicited no signs of cell damage as evaluated by trypan blue staining. After PMA-treatment the culture medium was exchanged with medium lacking PMA and the cells were allowed to recover for 24 h. For inflammatory activation cells were subsequently treated with 1 µg/ml lipopolysaccharide (LPS) (stock concentration was 100 mg/ml in demineralized water) and subjected to experiments. As a component of gram-negative bacteria LPS is a potent stimulator of inflammation and leads to secretion of TNF-α und IL-1-β [61], [62]. Signaling depends on the membrane-anchored receptor CD14 and Toll-like receptor (TLR) 4, which are functionally abundant on U937 cells [63], [64]. Cells differentiated and activated as described are referred to as U937-macrophages. The activated status of these cells was checked regularly with TNF-α-Enzyme Linked Immunosorbent Assay (ELISA) [65], [66].

### Cell Culture of Primary Human Macrophages

Primary human macrophages were isolated from blood donated by healthy volunteers (Blutspendedienst Zürich, www.zhbsd.ch) using “buffy coats”, a byproduct in the manufacturing of red blood cells and platelet concentrate from whole blood donation. All donors have signed an informed consent stating that blood or certain components may be anonymously used for research purposes (see www.zhbsd.ch) ). 200 ml blood containing 10 U/ml heparin was supplemented with 100 ml RPMI medium. Peripheral blood monocytic cells (PBMCs) were separated by density centrifugation (25 min, 500 g) through Ficoll-Paque (GE Healthcare, Glattbrugg, Switzerland). PBMCs were washed twice in RPMI and PBS (w/o Ca^2+^ and Mg^2+^) containing 0.5% bovine serum albumin each. Subsequently cells were suspended in RPMI with 10% fetal bovine serum, penicillin (100 U/ml) and streptomycin (100 mg/ml) and seeded at 3 x 10^6^ cells/ml in cell culture plates. After 1 h incubation under cell culture conditions, macrophageal cells had adhered to the flasks and non-adherent cells were removed. Cells were kept under culture conditions for three more days before they were subjected to experiments.

### Cell Culture of Human Osteoclasts

Osteoclasts were generated from human PBMCs. PBMCs were isolated from buffy coats which were obtained from the blood donation center Zurich (Blutspende Zürich, Schlieren, Switzerland). 15 ml of buffy coat was layered on 15 ml Ficoll Paque Premium (GE-Healthcare, Glattbrugg, Switzerland) in 50 ml-tubes and centrifuged for 30 min at 1500 g. The interface containing PBMCs was transferred to new 50 ml-tubes and washed 3 times with 50 ml ice cold PBS (resuspention and subsequent centrifugation for 15 min at 500 g). Before the last washing step all cells of one buffy coat were pooled. PBMCs were frozen in 90% FCS and 10% DMSO in a concentration of 30 x 10^6^ cells/ml and stored at –152°C. To generate osteoclasts, PBMCs were thawed and suspended in alpha-medium (Gibco, Invitrogen, Basel, Switzerland) containing 10% FCS, penicillin (100 U/ml), streptomycin (100 mg/ml), human M-CSF (25 ng/ml), human RANKL (50 ng/ml), human TGF-β (5 ng/ml) and dexamethasone (1 µM). Cells were seeded and kept under standard cell culture conditions for 10–12 days. Every 4 days half of the medium was exchanged. Starting from day 9, each day three representative wells of a 96-well plate with cells were fixed in 10% (v/v) formalin and stained using Toluidine Blue (0.1% for 1 min). Cells were inspected by light microscopy for osteoclasts morphology (three or more nuclei and a “ruffled border”). When cells deemed to be differentiated sufficiently (day 11+/−1) they were subjected to osteoclast resorption assay [67].

### Preparation of Primary Murine Microglia

Brains of 6 day old C57BL/6J mice were washed in 50 ml Hankś buffered saline (HBSS) and homogenized in 1 ml HBSS. 10 ml DMEM with 10% FBS, 2 mM glutamine, penicillin (100 U/ml) and streptomycin (100 mg/ml) (microglia-medium) was added. Cells were collected by 10 min centrifugation at 140 g, resuspended in 3 ml microglia-medium and seeded in poly-L-lysine-coated cell culture flasks (coating: 0.01 mg/ml, 0.05 ml/cm^2^, 1 h, 37°C). Cells were cultured in a humidified 5% CO2 atmosphere at 37°C. After 3–4 days cells were washed in HBSS and 5 ml fresh microglia-medium was added. After 3 more days the medium was changed, and microglia cells were detached from the cell culture flask by gentle tapping while astrocytes and fibroblast stayed adherent. Microglia cells were transferred to new poly-L-lysine-coated cell culture flasks, cultured for 3 days and then seeded in fresh microglia-medium in a concentration of 0.5 x 10^6^ cells/ml for experimental treatment.

### Osteoclast Resorption Assay

Osteoclasts were detached from culture dishes by incubation with Trypsin/EDTA (PAA, Cölbe, Germany) for 15 min at 37°C. Cells were resuspended in fresh medium and seeded on bovine cortical bone slices (Immunodiagnostic Systems, Frankfurt, Germany,) in a 96-well plate (0.25 x 10^6^/ml, 20 µl/well). Slices were inspected by light microscopy for bone resorbtion pits from 24 h after seeding onwards. When bone resorption pits were evident (48 h +/−24 after seeding) medium was removed and new medium with test substances (200 µl) was applied. 48–72 h later conditioned medium was collected. Osteoclast resorbtion activity was quantified by CrossLaps for Culture ELISA (CTX-1) (Immunodiagnostic Systems). ELISA was performed according to the manufacturer’s instructions. Conditioned medium was also subjected to MMP-9 activity ELISA (see separate section).

### Immunocytochemical Staining

U937-macrophages were differentiated into macrophageal cells on glass cover slips, activated and treated as previously described. Cells were fixed by 10 min incubation in 3.7% paraformaldehyde and blocked by subsequent 30 min incubation in 4% donkey-serum and 0.1% Triton X100 in PBS. After 3 washes cells were incubated in primary antibody against MMP-9 (Abcam ab 38904) 1∶100 in PBS 4% donkey-serum for 1 h at room temperature. Subsequently, cells were incubated with secondary Antibody (Alexa Fluor 555 donkey anti-rabbit IgG, Invitrogen) 1∶500 in PBS, 2% BSA, 0.1% Triton X100 for 1 h. In the second half hour phalloidin coupled to Alexa 488-dye (molecular probes) was added 1∶40 in order to stain the actin-cytoskelleton. Nuclei were stained by 5 min incubation in DAPI (Invitrogen) 1∶1000 in PBS. After 3 washes cells were mounted in prolong gold-mounting medium (molecular probes), and analyzed by fluorescent microscopy using the software “Application Suite 2.3.0 Advanced Fluorescence” (Leica).

### Cell Viability

The integrity of cell membranes was assessed by microscopic trypan blue exclusion technique viability was measured by MTT assay. U937-macrophages were differentiated in 96-well cell culture plates (2, 4, and 8 x 10^4^ cells/well) and treated with 2 or 4 µM WIN (or DMSO as vehicle control) for 24 h. Cells were supplied with 0.5 mg/ml MTT-tetrazolium-salt (3-(4,5-Dimethylthiazol-2-yl)-2,5-diphenyltetrazolium-bromide) and incubated for 3 h. Cell lysis was induced by adding 50 µl lysis-buffer (20% SDS, 50% N,N-dimethylformamid) per well and incubation at 37°C overnight. Absorption at 562 nm was measured and reductase enzyme activity was calculated as percentage of control after subtraction of blanks.

### MMP-9 Activity ELISA of Conditioned Medium

MMP-9 activity in conditioned medium of U937-macrophages, primary human macrophages and osteoclastic cells was quantified by the ELISA Kit Fluorokine E human Active MMP-9 (R&D Systems) according to the manufacturer’s instructions. All samples were diluted 1∶100 and 4-aminophenylmercuric acetate (APMA) was added to detect all potentially active MMP-9. Samples were measured in triplicates.

### Quantification of MMP-9 Protein in Conditioned Medium and BALF (Bronchoalveolar Lavage Fluid)

Conditioned medium of primary murine microglia and BALF were subjected to MMP-9 ELSA Kit Quantikine Mouse MMP-9 (R&D Systems) according to the manufacturer’s instructions. All samples were diluted 1∶2 and measured in triplicates.

### Western Blots Analyses

After exposure to the experimental conditions, U937-macrophages or primary human macrophages were scraped from the cell culture flasks and washed in ice cold PBS. Cells were suspended in lysis buffer (4 M urea, 0.5% sodium dodecyl sulfate (SDS), 62.4 mM tris(hydroxymethyl)aminomethane (Tris) pH 6.8, 10 µl/ml protease-Inhibitor-cocktail Set III (Calbiochem) and 1 mM phenylmethanesulfonylfluoride (PMSF)), incubated for 1 h on ice. Cells were sonified by 10 pulses, 1 second per pulse. Protein concentrations were measured using the BCA TM Protein Assay-Kit (Thermoscientific, Rockford, IL, USA). Cell culture supernatants were collected and concentrated 10-fold by vacuum centrifugation. Equal amounts of protein (15–30 µg) or 30 µl of concentrated conditioned medium were mixed with 4 x loading buffer (0.5 M Tris pH 6.8, 20% SDS, 50 mM sodium ethylenediaminetetraacetic acid (NaEDTA), 0.2% bromphenolblue, 10% Glycerol, 20% β-mercapthoethanol), denatured for 5 min at 95°C, and loaded on 8% SDS-gels (Separating gel: 466 mM Tris base pH 8.8, 8% polyacrylamid, 0.2% SDS, 0.75% ammonium persulfate (APS), 0.08% tetramethylethylenediamine (TEMED). Stacking gel: 466 mM Tris base pH 6.8, 5% polyacrylamide, 0.1% SDS, 0.75% APS, 0.08% TEMED). Elecrophoresis was carried out in 25 mM Tris base, 192 mM glycin, 0.1% SDS at 120 V. Subsequently, proteins were blotted onto cellulose nitrate membranes (Pierce, Thermo Fisher Scientific, Rockford, IL, USA) in transfer buffer (25 mM Tris base, 192 mM glycin, 20% methanol (v/v)). Membranes were rinsed in washing buffer (10 mM Tris, 150 mM NaCl, 0.05% tween) and blocked for 1 h in blocking buffer (washing buffer with 5% skimmed milk). Primary antibodies were diluted in blocking buffer and incubated overnight at 4°C or 3 h at room temperature. If not stated differently, MMP-9 was detected with mouse monoclonal anti-MMP-9 antibody (IM37L, Calbiochem, diluted 1∶2000 in washing buffer +5% BSA**)** that recognizes the aminoterminal end of MMP-9 or, when indicated, by a goat polyclonal antibody (sc-6840, Santa Cruz, diluted 1∶2000 in washing buffer +5% BSA) that recognizes the c-terminal end. Extracellular regulated kinase (ERK1/2) and phosphorylated ERK1/2 were recognized by rabbit polyclonal antibodies (9102 and 9101, Cell signaling, Boston, MA, USA, diluted 1∶1000 in washing buffer +5% BSA). Β-actin was detected with mouse Monoclonal Anti-β-Actin antibody (A544.1, Sigma, diluted 1∶10000 in blocking buffer). Membranes were washed and incubated at room temperature for 2 h with secondary antibodies (Odyssey: donkey anti-mouse IgG IRDye 680 (926–32222), donkey anti-mouse IgG IRDye 800 (926–32213), goat anti-rabbit IgG IRDye 680 (926–32221), goat anti-rabbit IgG IRDye 800 (926–32211), donkey anti-goat IgG IRDye 680 (926–32223), diluted 1∶10000 in blocking buffer). Membranes were washed three times and pictures were taken using Odyssey® Infrared Imaging System (LI-COR). Sizes of bands were estimated by comparison with a prestained molecular weight marker (Fermentas, Le Mont-sur-Lausanne, Switzerland) run on the same gel.

### Zymography

Gelatinolytic activity of MMP-9 in the conditioned medium was assessed by zymography. U937-macrophages and primary human macrophages were treated with WIN (or DMSO as vehicle control) for 24 h and conditioned medium was collected. Cells were removed by centrifugation at 500 g for 5 min. 8 µl of conditioned medium was mixed with non-reducing loading-buffer (0.5 M Tris pH 6.8, 20% SDS, 50 mM NaEDTA, 0.2% bromphenolblue, 10% glycerol) and loaded onto an 8% SDS-polyacrylamide gel (see SDS-PAGE) additionally containing 0.1% gelatine. Electrophoresis was performed as described. Renaturation was achieved by incubation in 50 mM Tris pH 7.4, 5 mM CaCl_2_, 1 µM ZnCl_2_, 2.5% Triton X100 for 30 min. Gelatinolytic bands were developed through incubation in 50 mM Tris pH 7.4, 5 mM CaCl_2_, 1 µM ZnCl_2_ for 24–48 h and subsequent staining with 0.5% Coomassie G250, 30% EtOH, 10% acetic acid and destaining with 30% ethanol, 10% acetic acid. Sizes of the clear bands were estimated by comparison with a prestained molecular weight marker (Fermentas) run on the same gel.

### Glycosidase Digestion

Cell lysates were prepared as described in the section *Western blot analyses*. Endoglycosidase H digestion: 10 µg protein were mixed with 80 µl Endoglycosidase H incubation buffer (1% Nonidet P-40, 25 mM EDTA, 50 mM Na-acetate, pH 5.5). Subsequently 0.4 µl β-mercapthoethanol was added, followed by denaturation at 95°C for 5 min. After addition of 1 µl PMSF, 0.5 µl proteinase inhibitor cocktail Set III and 1 µl (20 mU) endoglycosidase H (ROCHE, Mannheim, Germany), digestion took place at 37°C overnight. N-Glycosidase F digestion: 10 µg protein were mixed with 80 µl 25 mM Na-phosphat pH 7.5. Afterwards 0.2 µl β-mercapthoethanol was added, followed by denaturation at 95°C for 5 min. After addition of 1 µl proteinase inhibitor cocktail Set III, 0.45µl NP-40 and 4 µl (4 U) N-Glycosidase F (ROCHE) digestion took place at 37°C overnight. The samples were then subjected to SDS-PAGE.

### Quantitative Real-time Polymerase Chain Reaction (PCR) Analysis

Total RNA from U937-macrophages was isolated using Trizol reagent (Invitrogen, Basel, Switzerland) according to the manufacturer’s instructions and stored at −80°C. For cDNA-synthesis 1 µg of total RNA was transcribed with Reverse Transcription System-Kit (Promega, Wallisellen, Switzerland) using random hexameres. Quantification of mRNA levels of MMP-9 was achieved by quantitative real-time polymerase chain reaction using QuantiTect Primer Assay (QT00040040, Qiagen, Hilden, Germany) according to the manufactureŕs instructions. PCR reactions were run on a LightCycler® (Roche Applied Science) and mRNA level was calculated using delta-delta-CP-method with glycerinaldehyd-3-phosphat-dehydrogenase (GAPDH) as a reference gene (Qiagen, QT01192646) and control samples (treatment with only vehicle) as calibrator for calculation.

### Statistical Analysis

The comparison between two groups was performed with a t-test, while comparison between three or more groups was realised by analysis of variance and subsequent Newman-Keuls Multiple Comparison test. The analyses were carried out with the software “GraphPad Prism 3.0”, *p*<0.05 was considered statistically significant.

## Results

### WIN Treatment Decreased the Secretion and Activity of Matrix Metalloproteinase-9 in Inflammatory U937-macrophages

**Figure 1:**
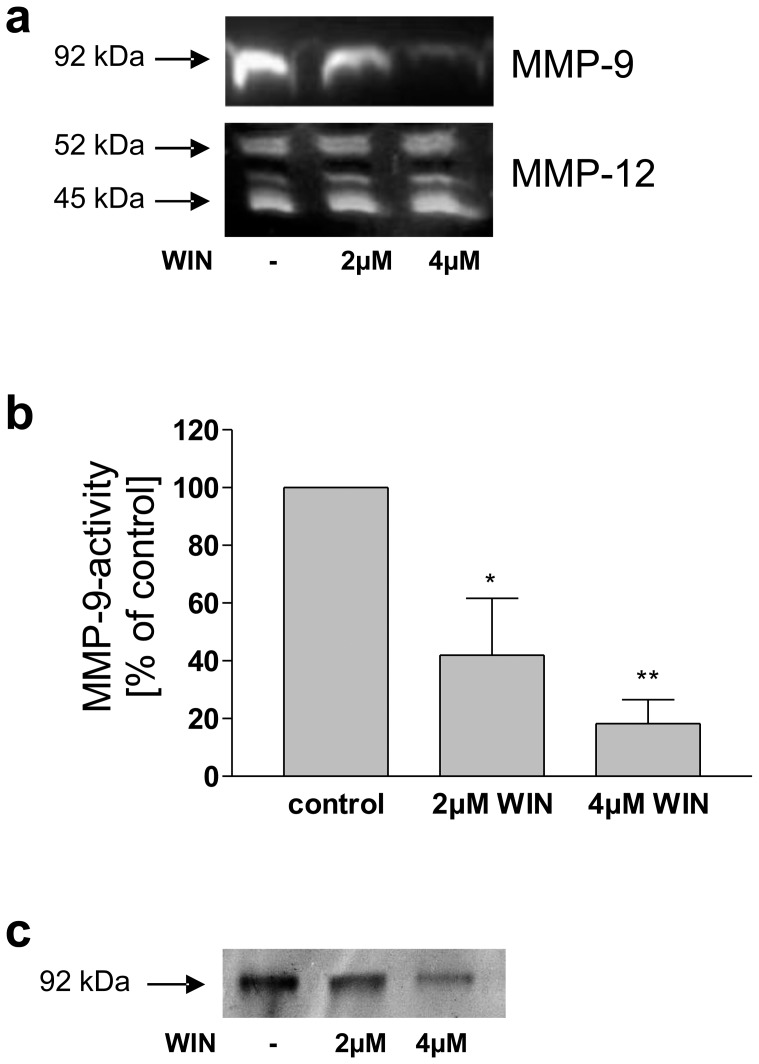
Treatment with WIN reduced secretion and activity of MMP-9 in macrophageal differentiated U937 cells. (a) Western blot analysis of conditioned medium using antibodies against MMP-9 and MMP-12. WIN-treatment resulted in a significant decrease of secreted MMP-9, whereas MMP-12 secretion was not affected. Control cells were treated with vehicle. The figure shows one representative analysis out of three. (b) MMP-9 activity-ELISA of conditioned medium. Upon treatment with 2 µM WIN a reduction of MMP-9 activity was observed, after treatment with 4 µM WIN the reduction was even stronger. Control cells were treated with vehicle. Data are shown as means +/− SD, n = 3. *p<0.05 vs. control, **p<0.01 according to Newman-Keuls Multiple Comparison test following ANOVA. (c) Zymography of conditioned medium. Gelatinolytic activity was strongly decreased by 2 and 4 µM WIN. The figure shows one representative analysis out of three.

As activated macrophages are a major source of MMP-9 in inflamed tissue, we first used PMA-differentiated and LPS-activated human U937 cells (U937-macrophages) as a model system. In this well-established model, PMA-differentiation leads to morphological and functional convergence to a macrophage-like phenotype, including a smooth surface, extended pseudopodia, cell cycle arrest and adherence to surfaces. Upon inflammatory stimulation with LPS, cells secrete pro-inflammatory mediators typical for activated macrophages, such as TNF-α, IL-1-β [61], [62], IL-6 [68] and the matrix metalloproteinases MMP-1, MMP-9 [69] and MMP-12 [70]. U937-macrophages were treated with 2 or 4 µM of WIN for 24 h. Subsequently, Western blot analysis was performed using antibodies against MMP-9. WIN treatment induced a decrease of secreted MMP-9 upon treatment with 2 µM, and an even stronger decrease upon treatment with 4 µM of WIN (fig. 1a). To investigate the specificity of WIN-induced inhibition of MMP-9 secretion, we also analyzed the secretion of MMP-12, the second most prominently expressed MMP in macrophages [71]. In contrast to MMP-9, the secretion of MMP-12 was not altered by WIN-treatment, which demonstrated that WIN does not generally inhibit MMP secretion. As the amount of MMP-9 protein was decreased, we investigated if this decrease is also valid at the level of activity. For this purpose, activity-ELISA and MMP-9 zymography were applied. The activity-ELISA displayed a strong down-regulation of MMP-9 activity to 42% (+/−20, n = 3) and 18% (+/−8, n = 3) of control, by 2 and 4 µM WIN respectively (fig. 1b). Accordingly, zymography demonstrated a decreased gelatinolytic activity after treatment with 2 and 4 µM WIN respectively (fig. 1c). To rule out toxicity of WIN, cell number, percentage of viable cells, and metabolic activity of U937-macrophages after WIN-stimulation were assessed. According to trypan blue staining, cell number and percentage of viable cell were not significantly altered by WIN-treatment. MTT-reduction was decreased to 94% (+/−13, n = 3) and 77% (+/−6, n = 3) upon 2 µM and 4 µM WIN respectively. In summary the data provides evidence, that WIN is not toxic on U937-macrophages in the applied concentrations. The observed MMP-9-bands of 92 kDa in Western blot analyses and zymography represent the latent form of MMP-9 which still contains the pro-sequence. Proteolytically activated MMP-9 (ca. 85 kDa) was not observed in our studies. This is in line with previous reports showing a presence of 92 kDa MMP-9 and an absence of the activated 85 kDa form and attributed to rapid dilution of MMP-9 and its potential activators in cell culture systems [72]. All in all, WIN-treatment specifically downregulated MMP-9 secretion and activity in inflammatory macrophageal cells.

### Inhibition of MMP-9 Secretion by WIN was Associated with an Intracellular Accumulation of Mature 92 kDa-MMP-9

**Figure 2:**
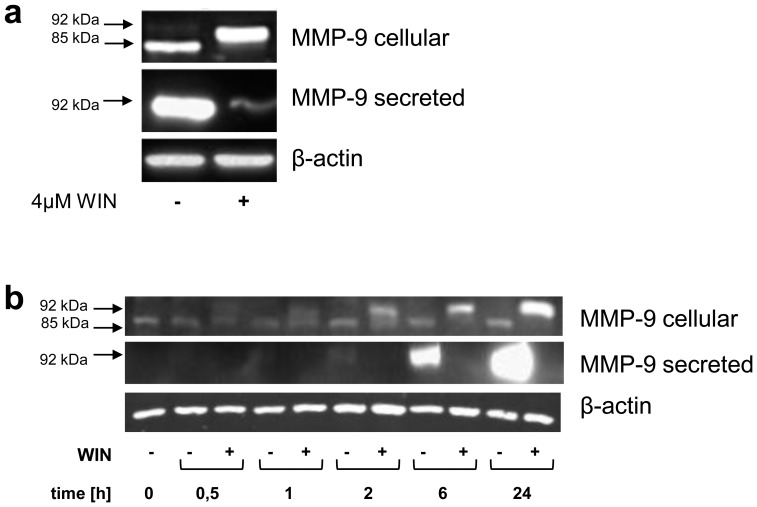
Inhibition of MMP-9 secretion was accompanied by an intracellular accumulation of a 92 kDa-MMP-9. Western blot analysis of cell lysates (MMP-9 cellular) and conditioned medium (MMP-9 secreted) of U937-macrophages treated with WIN using anti-MMP-9 antibody. Control cells were treated with vehicle. (a) Upon 24 h treatment with WIN, the amount of MMP-9 in the cell lysate increased, whereas the amount of secreted MMP-9 in the conditioned medium decreased. The size of MMP-9 in the cell lysate shifted from 85 kDa to 92 kDa. Control cells were treated with vehicle. The figure shows one representative analysis out of three. (b) Kinetic analysis of MMP-9 after WIN-treatment throughout 24 h. The inhibition of MMP-9 secretion was accompanied by an accumulation of intracellular 92 kDa-MMP-9. The intracellular 85 kDa-MMP-9 disappeared with time. The figure shows one representative analysis out of three.

**Figure 3:**
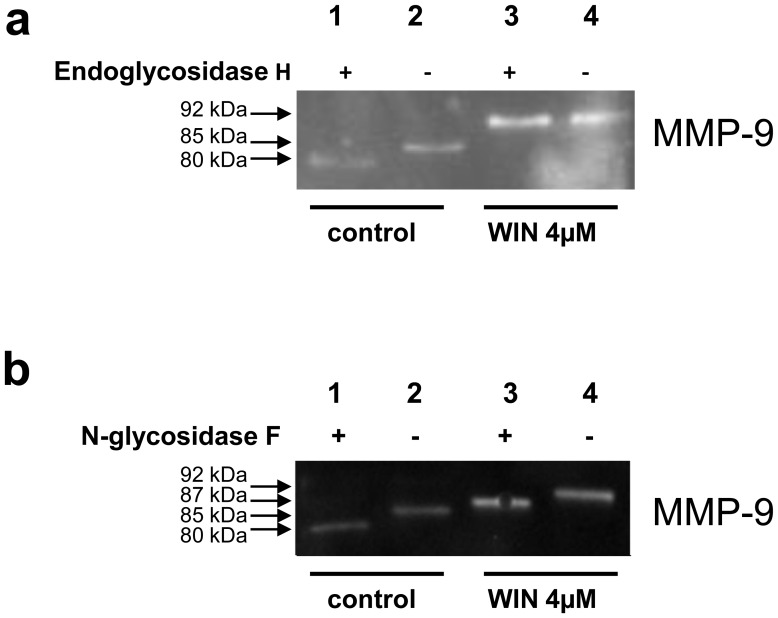
Glycosylation of intracellular 92 kDa-MMP-9 after WIN-treatment was different from the 85 kDa-MMP-9 in untreated U937-macrophages. The pictures show one representative analysis out of three. (a) Western blot analysis using anti-MMP-9 antibody of endoglycosidase H-digested cell lysates treated with or without WIN. The WIN-induced 92 kDa-MMP-9 was resistant to digestion, whereas the 85 kDa-MMP-9 from control cells loses 5 kDa upon digestion. (b) Digestion with N-glycosidase F resulted in a loss of 5 kDa in both MMP-9 forms.

Based on the observation that WIN strongly inhibited MMP-9-secretion in macrophages, we then investigated if MMP-9 biosynthesis was affected by WIN. We thus analysed MMP-9 in cell lysates of U937-macrophages after WIN-treatment with Western blot. Whereas we expected reduced amounts of intracellular MMP-9 upon WIN-treatment, surprisingly, the amount of intracellular MMP-9 was strongly enhanced (fig. 2a). Associated with the intracellular accumulation of MMP-9, we observed a band shift from 85 kDa to 92 kDa after WIN-treatment (fig. 2a). In a kinetic analysis of MMP-9 in WIN-treated macrophages, the intracellular 85 kDa-MMP-9 disappeared completely within 24 h, whereas the 92 kDa-MMP-9 appeared after 30 min as a weak band that increased with time (fig. 2b). 85 kDa is the size of MMP-9 that is usually described in cell lysates of U937 and other cells, it represents an immature form which is not yet fully glycosylated [73], [74]. 92 kDa is the size of fully glycosylated mature MMP-9 which is commonly found in conditioned medium [33], as also seen in our study (compare fig. 2a and fig. 2b). Because the 92 kDa-MMP-9 form in cell lysates appeared simultaneously to decreased secretion of MMP-9, we assumed that the MMP-9 secretion process was inhibited. This contributed to an intracellular accumulation of fully glycosylated mature MMP-9 inside the cells. To figure out whether the WIN-induced intracellular 92 kDa-MMP-9 form is indeed a mature form of MMP-9 (and therefore higher glycosylated than the 85-kDa MMP-9 form), we examined the glycosylation status of the intracellular 85 kDa and 92 kDa forms by means of Endoglycosidase H- and N-glycosidase F-digestion. Endoglycosidase-H does not affect fully glycosylated complex oligosaccharides, but specifically cleaves core oligosaccharide chains of the high-mannose and hybrid type [75]. For this reason endoglycosidase-H is a useful tool to distinguish fully glycosylated mature proteins from those holding unprocessed oligosaccharide chains. As demonstrated in fig. 3a, lanes 1 and 2, the intracellular 85 kDa-MMP-9 in the non-treated macrophages was endoglycosidase H-sensitive and had a size of 80 kDa after digestion. In contrast, the intracellular 92 kDa-MMP-9 from the WIN-treated macrophages was endoglycosidase H-resistant, this is consistent with the addition of complex carbohydrates (fig. 3a, lanes 3 and 4). We consequently investigated whether the size differences between the intracellular 85 kDa-MMP-9 and 92 kDa-MMP-9 was due to N-linked glycosylation. Both forms of MMP-9 were exposed to digestion by N-glycosidase F, which removes all N-linked oligosaccharide chains. Upon digestion, both MMP-9 forms lost 5 kDa of size (fig. 3b, lines 1–4). Taken together, the resistance of the intracellular 92 kDa-MMP-9 after WIN-treatment against endoglycosidase H digestion and the sensitivity of MMP-9 from untreated and WIN-treated macrophages against N-glycosidase F digestion suggest that the MMP-9 which accumulated after WIN-treatment was mature N-glycosylated MMP-9. Interestingly, the endogenous cannabinoids 2-Arachidonoylglycerol (2-AG) and *N*-arachidonoylethanolamide (Anandamid) (concentrations from 5 nM until 5 µM) did not induce an inhibition of secretion or an intracellular accumulation of MMP-9 (data not shown).

### WIN-treatment did not Alter Intracellular Distribution of MMP-9 in U937-macrophages

**Figure 4:**
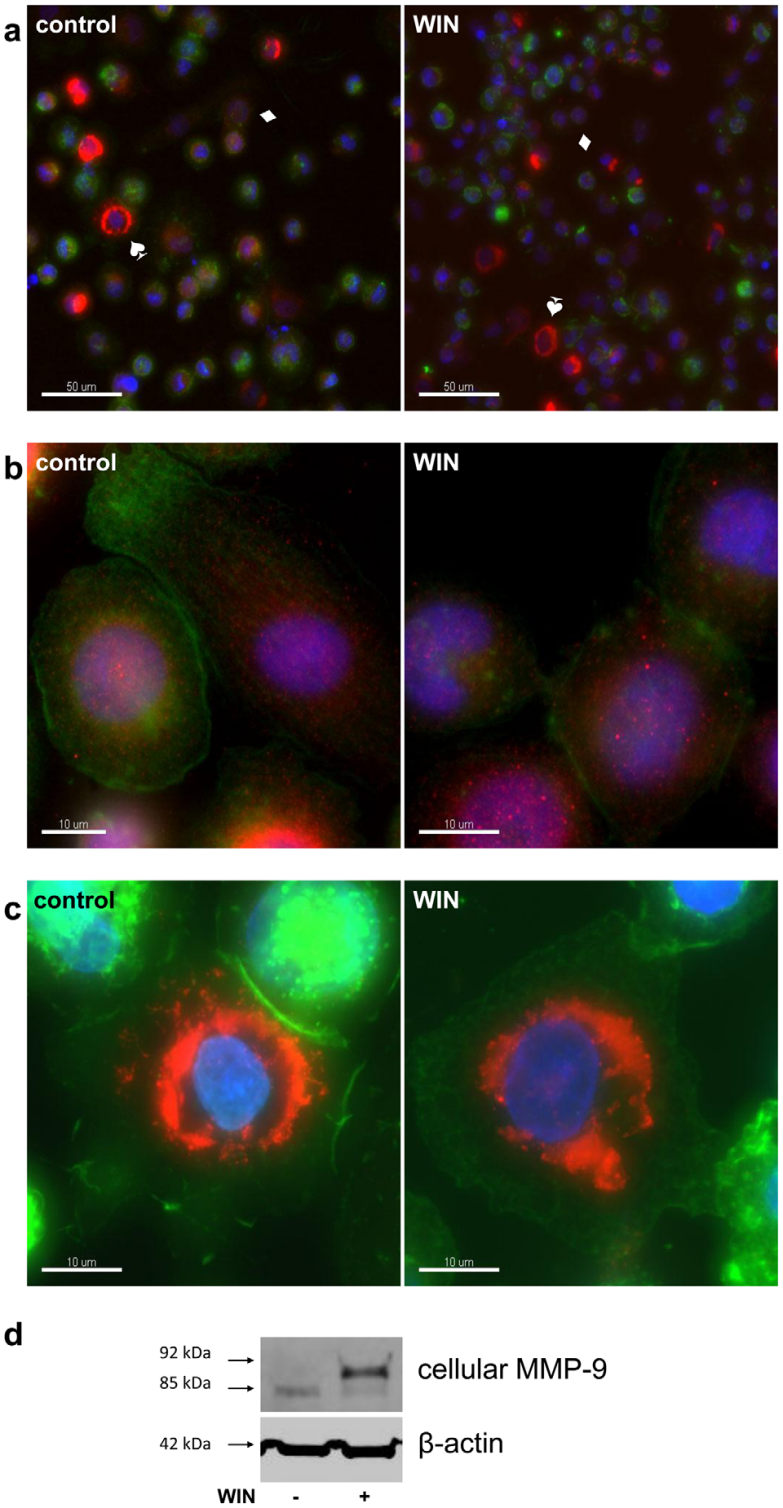
WIN did not alter the intracellular localization of MMP-9. Immunocytochemical staining of MMP-9 (red), F-actin (green) and nuclei (blue) of U937-macrophages which were treated for 24 h with 4 µM WIN or DMSO (control). The figure shows one representative analysis out of five. (a) Two types of MMP-9 expressing cells are observed in control cells and in WIN-treated cells, cells with a low MMP-9-signal in a vesicular pattern (♦), and cell with a brighter signal near the nucleus (♠). (b) Magnification of the cell type with MMP-9 in vesicular distribution. (c) Magnification of the cells type with perinuclear MMP-9-distribution. (d) Western blot analysis of the same samples using the same MMP-9 antibody as for immunocytochemistry (Abcam ab38904).

The accumulation of 92 kDa-MMP-9 seen by Western Blot analysis raised questions about the cellular localization of accumulated MMP-9. We investigated MMP-9 localization with immunocytochemical staining. The results are demonstrated in fig. 4, which shows that intracellular MMP-9 was localized in vesicles throughout the cytoplasm (fig. 4a and b) and in the perinuclear region (fig. 4a and c). However, WIN treatment did neither change the intracellular distribution pattern nor the amount of highly MMP-9 expressing cells (6.8% +/−0.6, n = 3, without WIN and 6.2% +/−0.9, n = 3, after WIN treatment). MMP-9 located at the surface of the cells was observed in neither of the cell types. The same samples were used for Western Blot analysis utilizing the same MMP-9 antibody as for the immunocytochemical staining, showing intracellular accumulation of a 92 kDa-MMP-9 as seen in the previous experiments (fig. 4d). Thus, immunocytochemical localization of M MP-9 revealed no changes in the intracellular distribution pattern of MMP-9 caused by WIN-treatment.

### Inhibited Secretion and Intracellular Accumulation of MMP-9 was Mediated by a Specific Binding Site Different from Classic Cannabinoid-receptors

**Figure 5:**
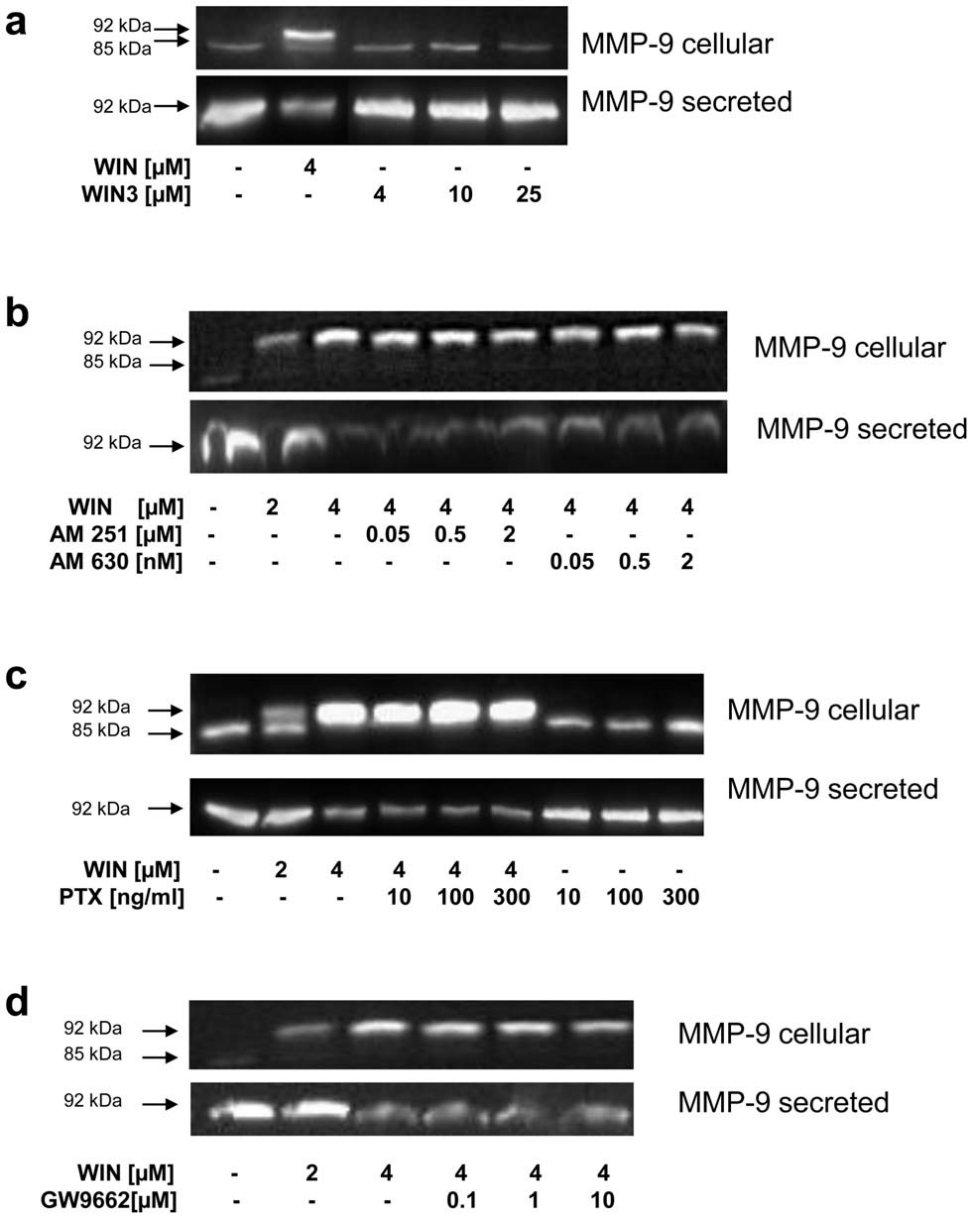
WIN-induced regulation of MMP-9 was mediated by a specific binding site, which is different from CB1, CB2, and PPARy, and independent from pertussis-toxin. Western blot analysis of cell lysates (MMP-9 cellular) and conditioned medium (MMP-9 secreted) of U937-macrophages treated with the receptor-inactive WIN-enantiomer S(–)-[2,3-Dihydro-5-methyl-3-[(4-morpholinyl)methyl]pyrrolo[1,2,3-de]-1,4-benzoxazinyl]-(1 naphthalenyl) methanone mesylat (WIN3), specific inhibitors for cannabinoid-receptors or pertussis toxin (PTX). Control cells were treated with vehicle. In each case the figure shows one representative analysis out of three. (a) Treatment with WIN3 demonstrated the specificity of the effect of WIN. (b) Inhibitors for CB1 (AM251) and CB2 (AM630) did not abolish the WIN-induced inhibition of secretion and intracellular accumulation of MMP-9. (c) Treatment with PTX did not abolish the WIN-induced effect. (d) Inhibition of PPARy with GW9662 had no influence on the effect of WIN on MMP-9.

In a next step we examined which receptor or binding site was responsible for the inhibition of the secretion and the increase of intracellular accumulation of MMP-9. For this we first tested if these effects are dependent on specific WIN binding or not, e.g. as an unspecific physical effect. Non-site-specific or physical effects such as changes in membrane fluidity should also be induced by a stereo-enantiomer of WIN. A stereo-enantiomer exhibits the same physical and chemical properties, but another steric specificity. For this reason we treated U937-macrophages with S(–)-[2,3-Dihydro-5-methyl-3-[(4-morpholinyl)methyl]pyrrolo[1,2,3-de]-1,4-benzoxazinyl]-1-naphthal-enyl)methanone mesylat (WIN3), which is a receptor-inactive enantiomer of WIN. As demonstrated in fig. 5a, 4 µM WIN3 was not able to reduce MMP-9 secretion, to induce an intracellular band shift or to lead to accumulation of MMP-9. This was also the case when WIN3 was used in much higher concentrations (10 or 25 µM). The data clearly indicates that WIN regulates MMP-9 secretion via a specific stereo-selective binding site.

To study whether this specific binding site is one of the classical cannabinoid receptors CB1 or CB2, these receptors were inhibited pharmacologically with AM251 and AM630, respectively. Surprisingly, inhibition of the receptors did not abolish or attenuate the inhibited secretion or the band shift and the intracellular accumulation of MMP-9 (fig. 5b). The CB-inhibitors alone did not have any effect. To investigate the involvement of G protein coupled receptors, we performed experiments with different concentrations of pertussis toxin. We found that the WIN-induced MMP-9 regulation was pertussis toxin-insensitive (fig. 5c). Another receptor which has shown to bind the cannabinoid ligands ajulemic acid and 2-Arachidonoylglycerol (2-AG) is the peroxisome proliferator-activated receptor-γ (PPARγ) [76]. This receptor belongs to the “nuclear hormone receptor family of ligand-dependent transcription factors” and it mediates signals in adipocyte-differentiation, glucose-metabolism, and immune regulation [77]. Experiments with 2-chloro-5-nitrobenzanilide (GW9662), a potent PPARγ antagonist, demonstrated that WIN-induced inhibition of secretion and intracellular accumulation of MMP-9 was independent of PPARγ (fig. 5d).

### WIN-induced Intracellular Accumulation and Inhibition of MMP-9 Secretion was Mimicked by the TRPV1 Inhibitor Capsazepine and Antagonized by the TRPV1 Agonist Capsaicin

**Figure 6:**
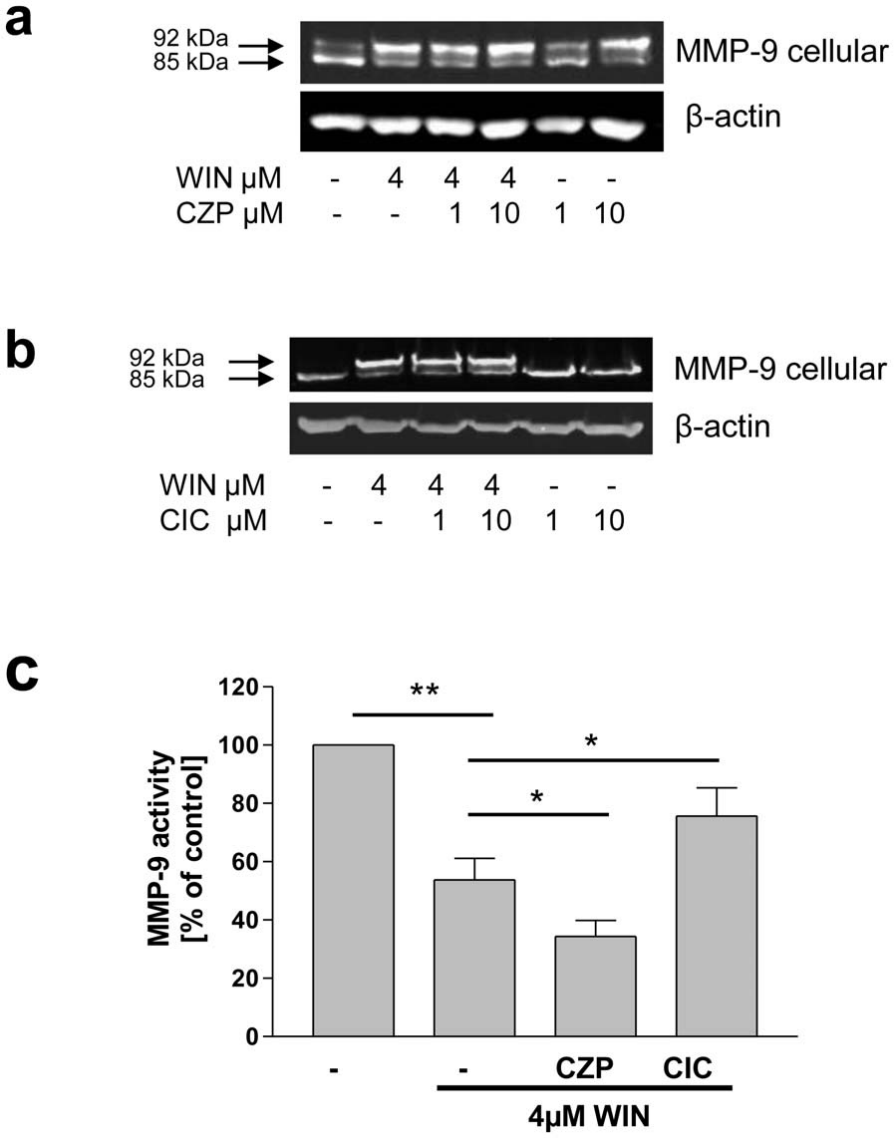
TRPV1 was involved in inhibition of secretion and intracellular accumulation of MMP-9 upon WIN-treatment. (a,b) Western blot analyses of U937-macrophage cell lysates (MMP-9 cellular) using MMP-9 antibody. (a) Treatment with the TRPV1 antagonist capsazepine (CZP) enhanced the WIN-induced size shift of MMP-9 from 85 to 92 kDa when given parallel to WIN and mimicked this effect when administered separately. The figure shows one representative analysis out of three. (b) Treatment with the TRPV1 agonist capsaicin (CIC) antagonized the WIN-induced size shift while it exhibited no effect when given alone. The figure shows one representative analysis out of three. (c) MMP-9 activity–ELISA of conditioned medium. The WIN-induced decrease of MMP-9 activity was intensified by CZP (10 µM), and antagonized by CIC (10µM). Data are shown as means +/− SD, n = 3. **p<0.001 *p<0.1 according to Newman-Keuls Multiple Comparison test following ANOVA.

In the search for the functional WIN-binding site we subsequently wanted to assess a possible involvement of TRPV1 in the WIN-induced inhibition and intracellular accumulation of MMP-9. TRPV1 has been shown to mediate WIN-induced effects in neuronal and immunological functions [78], [79]. For this reason, we performed experiments using pharmacological inhibition and stimulation of TRPV1. Inhibition of TRPV1 by capsazepine (CZP) enforced a similar band shift of MMP-9 as the incubation with WIN (fig. 6a), whereas we could not detect an additive effect of WIN and CZP. Activation of TRPV1 by the agonist capsaicin (CIC) resulted in a partial antagonisation of the WIN-induced band shift of MMP-9 (fig. 6b). Measurement of MMP-9 activity in the conditioned medium revealed that CZP enhanced the WIN-induced downregulation of MMP-9 secretion, whereas CIC reduced ameliorated this effect (fig. 6c). CZP significantly increased the decline of MMP-9-activity after WIN treatment from 55% (+/−8, n = 3) to 34% (+/−6, n = 3) compared to control cells, whereas CIC antagonized the WIN-induced effect and reduced the decline of MMP-9-activity significantly from 55% (+/−8, n = 3) to 75% (+/−9, n = 3) (fig. 6c). Thus, ion channel TRPV1 represents a possible binding site of WIN by which MMP-9 secretion is inhibited and intracellular accumulation is promoted.

### Intracellular Accumulation of MMP-9 was not Mediated via Rho Signaling

In a next step we looked for a possible mechanism by which WIN might exert its inhibiting effect on MMP-9 secretion. It has been demonstrated that Rho/ROCK signaling is involved in the regulation of MMP-9 secretion [80], [81], [82]. We incubated U937-macrophages with the Rho-inhibitor Y27632 to assess if Rho signaling is essential for the secretion of MMP-9 in our model system. We detected a slight decrease of MMP-9 secretion by Y27632, but no effect on intracellular MMP-9 accumulation (data not shown). We reckon that Rho signaling may contribute to the regulation of MMP-9 secretion in our model system, but via different mechanisms than the WIN-induced intracellular accumulation of MMP-9.

### Transcriptional Regulation of MMP-9 upon WIN Treatment Involved ERK-Phosphorylation

**Figure 7:**
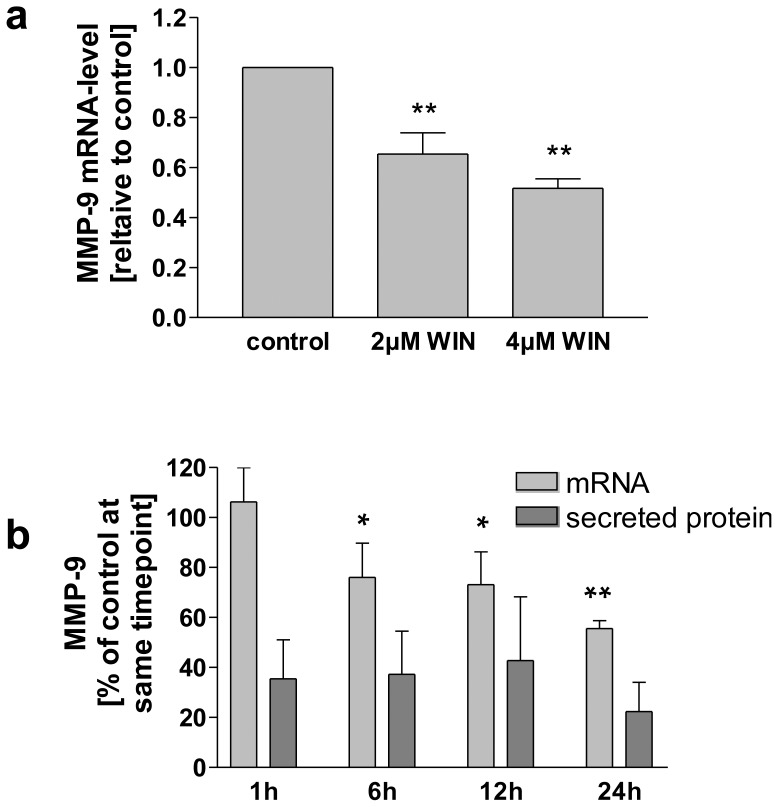
WIN down-regulated MMP-9 mRNA in U937-macrophages. (a) Quantitative real-time PCR of MMP-9 mRNA. 2 µM and 4 µM WIN decreased MMP-9 mRNA to 66% and 55% respectively. Data are shown as means +/− SD n = 3. **p<0.01 vs. control according to Newman-Keuls Multiple Comparison test following ANOVA. (b) Comparison of MMP-9 mRNA and secreted protein, assessed by real-time PCR (n = 3) and densitometry of Western blot analyses (n = 2). Values of mRNA and secreted MMP-9 Protein are given as percentage of the amounts that were measured in untreated cells at the corresponding time point. Secretion of MMP-9 protein was already decreased to 40% after 1 h, and remained in this range for 24 h. In contrast, no mRNA decrease was observed after 1 h, mRNA level decreased steadily to 55% after 24 h. Data are shown as means +/− SD, *p<0.05, **p<0.01 vs. 1 h according to Newman-Keuls Multiple Comparison test following ANOVA.

In the following we investigated if transcriptional regulation plays a role in intracellular MMP-9 protein accumulation upon WIN treatment. We performed quantitative real-time measurements of MMP-9 mRNA expression. As demonstrated in fig. 7a, MMP-9 mRNA was downregulated to a ratio of 0.653, (+/−0.085, n = 3) and 0.517, (+/−0.037, n = 3) by 2 µM WIN and 4 µM WIN compared to the control sample. Because downregulation of MMP-9 mRNA expression was associated with intracellular accumulation of MMP-9 protein, we assumed a negative feedback effect of MMP-9 protein on MMP-9 mRNA expression. Thus, we compared the kinetics of MMP-9 mRNA down-regulation with the down-regulation of MMP-9 protein secretion (fig. 7b). Levels of mRNA and secreted MMP-9 protein are given as percentage of the amounts that were measured in untreated cells at the corresponding time points. In fig. 7b it is demonstrated that MMP-9 mRNA expression level after 1 h WIN-treatment was 106% (+/−13.6, n = 3) and decreased afterwards to 56% (+/−3.0, n = 3) within 24 h. In contrast, the level of secreted protein after 1 h was already decreased to 36% (+/−15, n = 3) compared to the appropriate controls, and the degree of down-regulated secretion did not changed much during 24 h (37% (+/−17, n = 3) after 6 h, 43% (+/−26, n = 3) after 12 h, 22% (+/−12, n = 3) after 24 h). Thus, mRNA down-regulation occurred with a time delay compared to downregulation of MMP-9 secretion, suggesting a negative feedback mechanism on MMP-9 mRNA expression by the disturbed secretion and drastic intracellular accumulation (see fig. 2 and fig. 3).

**Figure 8:**
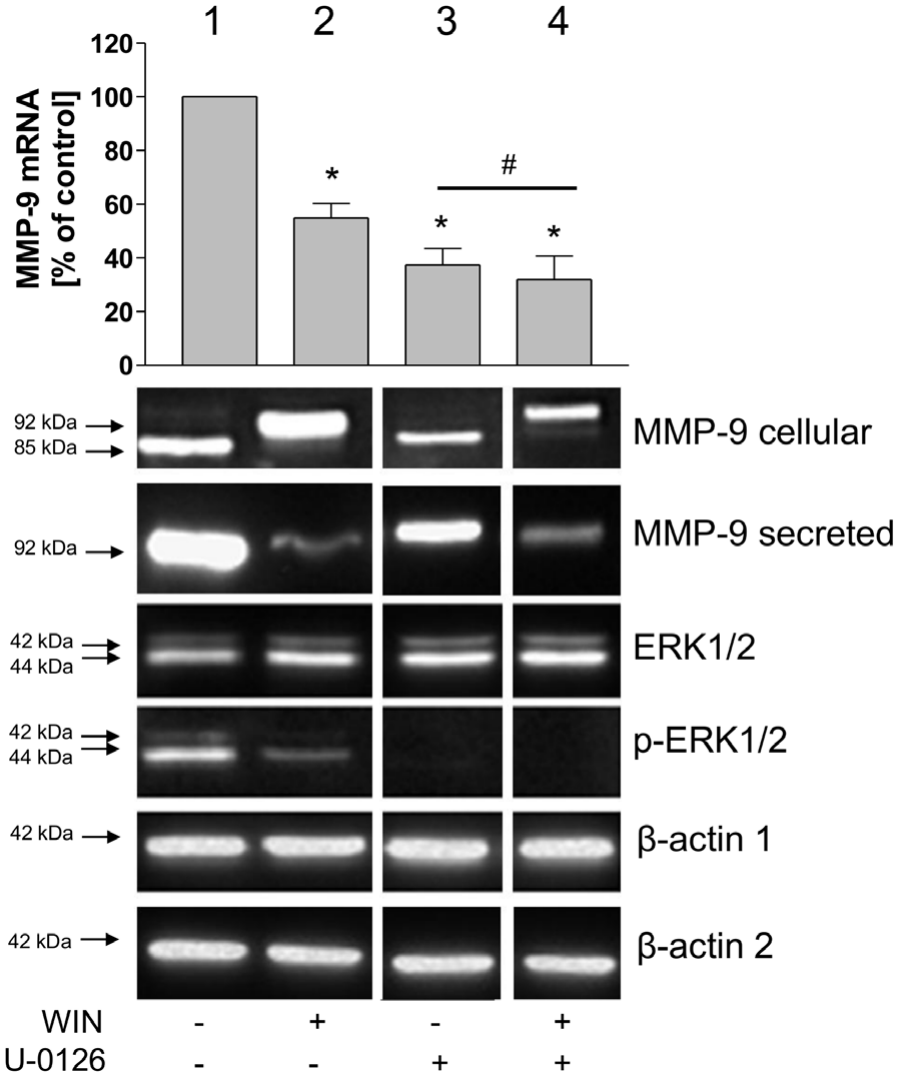
Dephosphorylation of ERK1/2 is involved in the WIN-induced down regulation of MMP-9 mRNA, but not in the effect of WIN on MMP-9 protein. MMP-9 quantitative Real-time PCR (bar chart) and Western blot analyses of intracellular (MMP-9 cellular) and secreted (MMP-9 secreted) MMP-9 and for phosphorylated (pERK1/2) and unphosphorylated ERK1/2. The figure shows one representative analysis out of three. U937-macrophages were treated with WIN (4 µM) or the pharmacological ERK1/2 phosphorylation inhibitor U-0126 (50 µM). Lane1: control; lane 2: WIN-treatment; Lane 3: U0126-treatment; lane 4: WIN + U0126-treatment. WIN treatment resulted in a dephosphorylation of ERK associated with inhibition of secretion, intracellular accumulation and decrease of MMP-9 mRNA (lanes 1 and 2). Inhibition of ERK1/2 phosphorylation with U-0126 decreased the level of MMP-9 mRNA significantly, but did not affect MMP-9 protein (lane 3). Treatment with WIN and U-0126 together did not decrease MMP-9 mRNA further than caused by U-0126 alone (lines 3 and 4). Bar chart: Data are shown as means +/− SD n = 3. *p<0.01 vs. control, ^#^p>0.05 according to Newman-Keuls Multiple Comparison test following ANOVA. Western blot: ß-actin 1 is from the same blot as p-EKR1/2, ß-actin 2 is from the same blot as MMP-9 and ERK1/2. Originally, the blots contained more samples. In order to arrange the figure for comparison with mRNA quantification, blots were cut and rearranged. Original lanes can be seen in Figure S1.

Recently it has been shown that dephosphorylation of ERK1/2 leads to an inhibition of MMP-9 transcription [35], [83]. We investigated the hypothesis that the phosphorylation status of ERK1/2 could be involved in the observed WIN-induced reduction of MMP-9 mRNA. As demonstrated in fig. 8, column 1 and 2, 4 µM WIN contributed to a profound dephosphorylation of ERK1/2 accompanying the inhibition of MMP-9 secretion, intracellular MMP-9 protein accumulation and a decrease of MMP-9 mRNA. To investigate if dephosphorylation of ERK1/2 was responsible for down regulation of MMP-9 mRNA in our system, we performed experiments using the ERK1/2-phosphorylation inhibitor U-0126. As demonstrated in fig. 8, column 3, dephosphorylation diminished the amount of MMP-9 mRNA to 37% (+/−6, n = 3), which was a significantly lower than after WIN-treatment alone, which down-regulated MMP-9 mRNA to 55% (+/−5, n = 3). Parallel treatment with U-0126 and WIN caused a non-significant further decrease of MMP-9 mRNA to 32% (+/−9, n = 3) (*P* = 0.17). Dephosphorylation of ERK1/2 by U-0126 was associated with a slight reduction of MMP-9 secretion, but no intracellular accumulation of MMP-9 protein was detected (fig. 8, column 4). To sum up, ERK1/2 was dephosphorylated by WIN treatment and pharmacological inhibition of ERK1/2 phosphorylation decreased MMP-9 mRNA expression, but did not affect MMP-9 maturation. For this reason we suggest that ERK1/2 phosphorylation participated primarily in MMP-9 mRNA down-regulation, but secondary to intracellular accumulation of MMP-9 protein.

### Inhibited Secretion and Intracellular Accumulation of MMP-9 in WIN-treated Inflammatory Primary Human Monocytes

**Figure 9:**
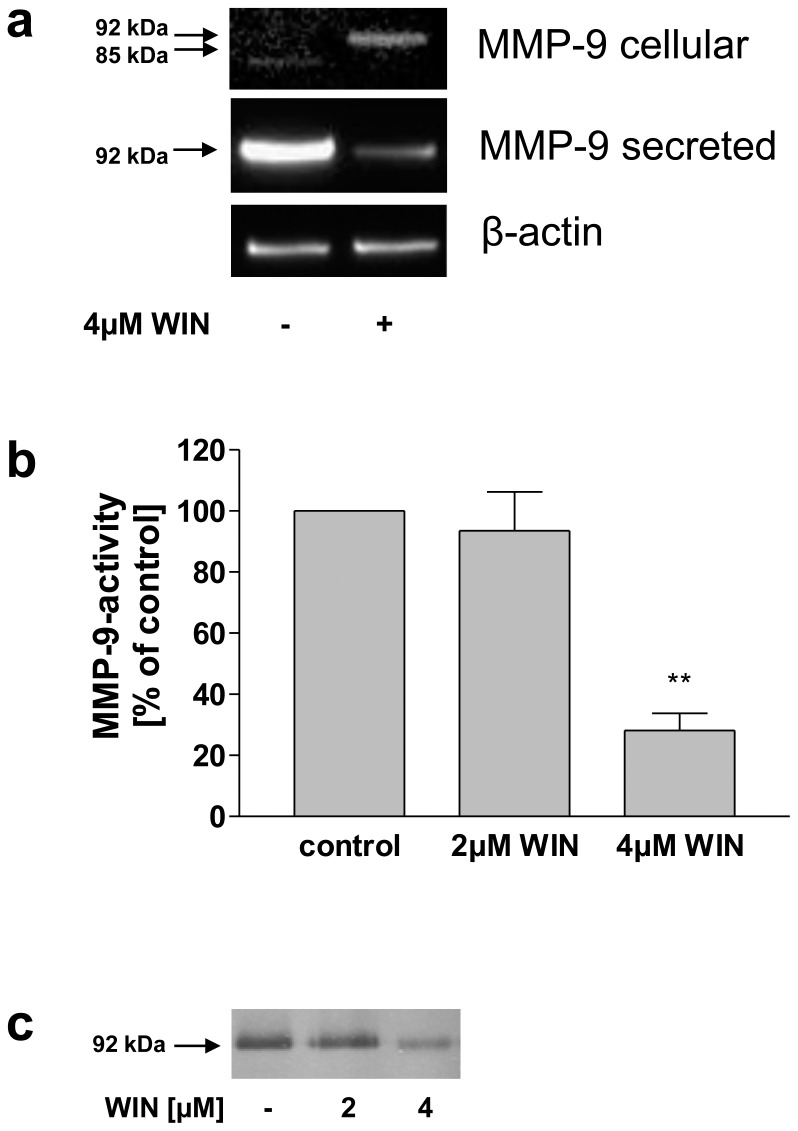
Inhibition of MMP-9 secretion and activity and intracellular accumulation of MMP-9 in WIN-treated activated primary peripheral monocytes. (a) Western blot analysis of cell lysates (MMP-9 cellular) and conditioned medium (MMP-9 secreted) using anti-MMP-9 antibodies. The figure shows one representative analysis out of three. WIN inhibited MMP-9 secretion and induced an intracellular accumulation of 92 kDa MMP-9. (b) MMP-9 activity-ELISA of conditioned medium. Upon treatment with 2 and 4 µM WIN, a concentration-dependent reduction of MMP-9-activity was observed. Data are shown as means +/− SD n = 3. **p<0.01 vs. control according to Newman-Keuls Multiple Comparison test following ANOVA. (C) Zymography of conditioned medium. Gelatinolytic activity was inhibited by WIN-treatment. The figure shows one representative analysis out of three.

To investigate whether the WIN-induced effect on MMP-9 regulation is also present in primary human cells of the monocyte-macrophages system, we performed the key experiments in LPS-stimulated primary peripheral monocytes isolated from human blood. Western blot analyses of conditioned medium and cell lysates revealed that WIN inhibited MMP-9 secretion and induced a band shift of the intracellular MMP-9 protein from 85 kDa to 92 kDa as observed in U937-macrophages (fig. 9a). MMP-9 activity-ELISA demonstrated that MMP-9 activity in the conditioned medium was reduced significantly by 4 µM WIN to 28% (+/−9, n = 3), whereas 2 µM WIN had no significant effect (fig. 9b). Accordingly, a decreased gelatinolytic activity after treatment with 4 µM WIN was observed in zymography (fig. 9c). Therefore, inhibited secretion and intracellular accumulation of 92 kDa-MMP-9 was also present in WIN-treated inflammatory primary human monocytes.

### WIN-induced MMP-9 Regulation in Human Primary Osteoclasts, but not in Primary Murine Microglia Cells

**Figure 10:**
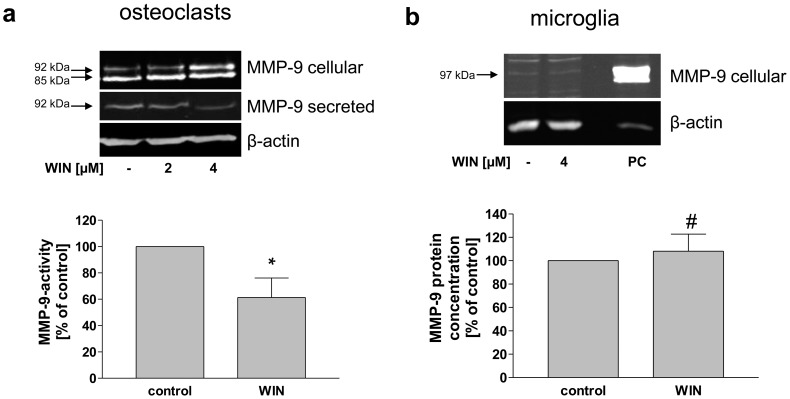
Inhibition of MMP-9 secretion and activity and intracellular accumulation of MMP-9 in WIN-treated osteoclasts, but not in microglia. (a) Western blot analysis of cell lysates (MMP-9 cellular) and conditioned medium (MMP-9 secreted). using anti-MMP-9-antibody and MMP-9-activity ELISA of conditioned medium (bar chart) from osteoclasts. Upon WIN treatment (4 µM), the amount of intracellular 92 kDa-MMP-9 was enhanced, while the amount of secreted MMP-9 and the activity of MMP-9 in the conditioned medium was decreased. (b) Western blot analysis of cell lysates (MMP-9 cellular) using anti-MMP-9-antibody and MMP-9 ELISA of conditioned medium (bar chart) from primary microglia. Size and amount of intracellular MMP-9 were not changed after WIN-treatment (4 µM). The amount of MMP-9 in the conditioned medium increased insignificantly. PC = positive control (U937 macrophages). The figure shows one representative analysis out of three. Data are shown as means +/− SD n = 3. *p<0.05, ^#^p>0.05 vs. control according to unpaired t test.

After we identified the regulation of MMP-9 maturation and secretion by a WIN-sensitive site in a macrophageal differentiated human cell line (U937-macrophages) and in primary human monocytes, there is a possibility that this regulatory principle is also present in other cell types of the monocyte-macrophage system. For this reason we also studied the effect on the bone- and brain-resident macrophages, osteoclasts and microglial cells. As demonstrated in fig. 10a, WIN induced an accumulation of intracellular 92 kDa-MMP-9 and a decrease of the secreted MMP-9 in osteoclasts as it was observed in macrophageal differentiated U937- and primary macrophageal cells. Secreted MMP-9-activity was reduced significantly to 61% (+/−29, n = 4) upon WIN-treatment. In contrast we did not detect a clear MMP-9 band in Western blot-analysis of primary murine microglia cells. There was no change in size or quantity after treatment with WIN. Quantification of secreted MMP-9 by ELISA revealed no significant changes upon WIN-treatment in primary microglial cells (fig. 10b).

### Bone Resorption Activity of Osteoclastic Cells was Decreased Upon WIN-treatment and Dependent on TRPV1

**Figure 11:**
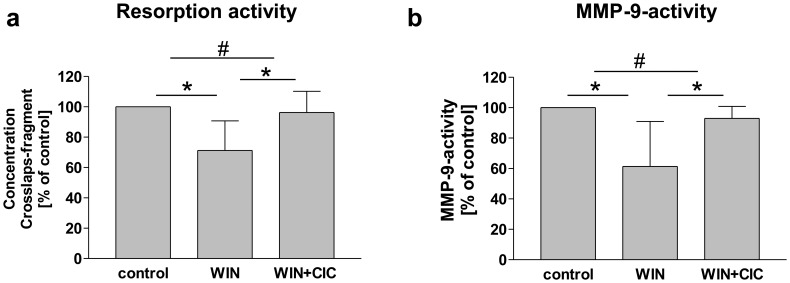
WIN reduced bone resorption and MMP-9-activity in a capsaicin sensitive manner. (a) Measurement of resorption activity of osteoclasts using crosslaps-ELISA of conditioned medium. Treatment with WIN (4 µM) reduced the osteolytic activity compared to control cells (vehicle treated). Additional treatment with capsaicin (CIC) antagonized this decrease. Data are shown as means +/− SD, n = 5. (b) MMP-9-activity-ELISA of conditioned medium of osteoclasts. Treatment with WIN (4 µM) decreased MMP-9-activity significantly compared to control cells (vehicle treated) and this decrease was antagonized by parallel treatment with CIC. Data are shown as mean +/− SD, n = 5. *p<0.05, ^#^p>0.05 according to Newman-Keuls Multiple Comparison Test following ANOVA.

After detecting WIN-sensitive MMP-9 maturation and secretion in primary osteoclasts, and after establishing that MMP-9 is a key protein in osteoclast activity and bone resorption [39], [41], we observed if WIN-treatment was capable of reducing bone resorption by osteoclasts. We performed an *in vivo*-like bone resorption assay (Crosslaps-ELISA) as a model system to estimate the functional relevance of the WIN-induced inhibition of MMP-9. According to our hypothesis, quantification of bone resorption activity revealed that WIN-treatment resulted in a significant down-regulation of bone resorption to 71% (+/−19, n = 5) compared to control cells (vehicle treated). In the following, we tested the possibility that the decreased MMP-9 secretion and subsequent reduction of bone resorption by osteoclasts were based on TRPV1 activation, as revealed for U937-macrophages (see fig. 6). As demonstrated in fig. 11, reduced bone resorption and MMP-9 secretion after WIN treatment were antagonized by the TRPV1 agonist capsaicin (CIC). The reduced bone resorption was reversed ameliorated significantly from 71% (+/−19, n = 5) to 96% (+/−14, n = 5) (fig. 11a), and the reduced MMP-9 activity in conditioned medium was restored from 61% (+/−29, n = 4) to 93% (+/−8, n = 4) (fig. 11b).

### The synthetic Cannabinoid WIN 55,212 2 (WIN) Decreased the Amount of MMP-9 Secreted by White Blood Cells in Bronchoalveolar Lavage Fluid (BALF) in a Murine Model of Smoke-induced Lung Inflammation

**Figure 12:**
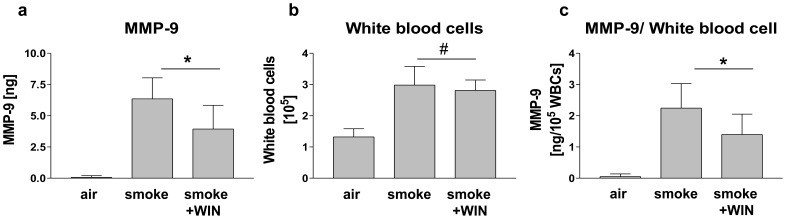
Treatment with WIN reduced MMP-9 protein in bronchoalveolar lavage fluid (BALF) of mice with smoke-induced lung inflammation. Mice were exposed to air, cigarette smoke (smoke), or cigarette smoke plus i.p. treatment with 5 mg/kg/d WIN (smoke + WIN). (a) MMP-9 protein was measured by ELISA in BALF. Cigarette smoke-exposure enhances the MMP-9-content of BALF. I.p application of WIN during cigarette smoke-exposure reduced MMP-9 in BALF. (b) Number of white blood cells (WBCs) in BALF measured by haemocytometry. Cigarette smoke-exposure enhanced the content of WBCs in BALF significantly. I.p. application of WIN during cigarette smoke-exposure did not alter the number of WBCs. (c) Ratio of MMP-9/10^5 ^WBCs. The amount of MMP-9 per WBC decreased upon i.p. application of WIN significantly. Data are shown as means +/− SD, n = 7 (air) n = 8, (smoke), n = 9 (smoke+WIN). *p<0.05, ^#^p>0.05 according to Newman-Keuls Multiple Comparison test following ANOVA.

To look into wheather the *in vitro* observed suppressive effect of WIN on MMP-9 secretion is also valid *in vivo*, we applied a murine model of cigarette smoke-induced lung inflammation. Lung inflammation was achieved by cigarette-smoke-exposure over 4 days (control animals received air instead of smoke), and the mice were treated with WIN (5 mg/kg, i.p.) or vehicle daily. MMP-9 was then measured in bronchoalveolar lavage fluid (BALF) with ELISA. As the main source for MMP-9 and as an indicator for the strength of inflammation white blood cells (WBC) were quantified in BALF. In the BALF of animals that received air a total amount of 0.07 ng (+/−0.13 n = 7) MMP-9 was measured, whereas animals with smoke-induced lung inflammation had a content of 6.36 ng (+/−1.68, n = 8). I.p. injection of WIN led to a decrease of MMP-9 in BALF to 3.94 ng (+/−1.90, n = 9) (fig. 12a). The total number of WBCs in BALF was increased by smoke-exposure from 1.321 x 10^5^ (+/−0.26 n = 7) in the air-treated group to 2.980 x 10^5^ (+/−0.601, n = 8) in the smoke-group. I.p. WIN-treatment did not alter the amount of WBCs significantly (2.812 x 10^5^ (+/−0.334, n = 9)) (fig. 12b). Nevertheless, to rule out the possibility that the decrease in MMP-9 was due to changes in WBC cell number, the amount of MMP-9 was calculated as ratio between MMP-9 and WBCs. This ratio decreased significantly from 2.25 ng/10^5 ^WBCs (+/−0.2782, n = 8) to 1.40 ng/10^5 ^WBCs (+/−0.2168 n = 9) as a consequence of WIN treatment (fig. 12c). Thus, administration of the cannabinoid receptor agonist WIN was capable of inhibiting MMP-9 release *in vivo* in a mouse model of lung inflammation.

Taken together, we demonstrated that binding of the cannabinoid-receptor agonist WIN to a stereo-selective, specific binding site in cells of the monocyte-macrophage-system induced a significant disturbance of MMP-9 processing and secretion, which subsequently down-regulated MMP-9 mRNA expression. This downregulation probably occurred via ERK1/2-phosphorylation-dependent pathway. We suppose an involvement of TRPV1, but other still unidentified sites present further possibilities. Downregulation of MMP-9 activity was demonstrated in lung inflammation in an *in vivo* murine model and in *in-vivo-like* bone tissue cultures with active osteoclasts. They are examples of possible functional consequences of MMP-9 downregulation within the monocyte-macrophage-system.

## Discussion

The collagenase MMP-9 constitutes a crucial element of inflammation and it is causally involved in severe tissue destruction during inflammatory conditions including inflammatory bowel disease [47], vascular disease [46], lupus erythematosus, Sjögren’s syndrome, sclerodermia, polymyositis, multiple sclerosis [48] and COPD [84]. Therefore, inhibition of MMP-9 secretion or activity is considered a promising therapeutic target during inflammatory diseases. Many inhibitors have been developed and they have been tested *in vivo* [26], [29]. In our study, we present evidences that MMP-9 maturation and secretion can be drastically mitigated by the cannabinoid receptor agonist WIN55,212-2. We further suggest that this anti-inflammatory action is mediated by TRPV1 receptors. Finally, we found that the cannabinoid receptor agonist WIN55,212-2 represents a potent tissue protective drug which reduced MMP-9 activity in lung inflammation *in vivo* and osteoclast-mediated bone destruction in an *in vivo*-like model system.

Anti-inflammatory properties of WIN have been described in previous *in vivo* studies: Berdyshev et al. found that intranasal application of WIN reduced TNF-α concentration in BALF in a mouse model of LPS-induced inflammation [85]. In an arteriosclerosis model of the apolipoprotein E-knockout (ApoE(−/−)) mouse, administration of WIN reduced macrophageal invasion in plaque lesions, decreased pro-inflammatory gene expression and NF-kappaB activation in aortic tissues and reduced the size of atherosclerotic lesions in the aorta root [58]. In experimental autoimmune encephalomyelitis (EAE), treatment with WIN reduced the inflammatory infiltration of brain tissue with T cells and microglia/macrophages and reduced axonal degeneration and demyelination [57].

In our study we found that WIN decreased the secretion of MMP-9 protein and enzymatic activity *in vitro* in several cell types of the human peripheral monocyte-macrophage-system, namely: macrophageal differentiated U937 cells, primary peripheral monocytes and primary osteoclasts. In murine primary microglia, we did not detect an effect of WIN on MMP-9 secretion, whereas our *in vivo* experiments using a mouse model of smoke-induced lung inflammation demonstrated a reduced MMP-9 secretion after WIN treatment in BALF. Thus, the question if microglial cells respond to WIN with downregulation of MMP-9 secretion, remains open and can only be solved in experiments with primary human microglia. An influence of WIN on the regulation of MMP-9 was described for cancer cell lines [54], [86], but so far not for cells of the immune system. Comparable concentrations of WIN were reported to inhibit macrophageal secretion of oxidized low-density lipoprotein-induced TNF-α and reactive oxygen species in RAW264.7 macrophages, primary murine peritoneal macrophages [87], and of LPS-induced nitric oxide in RAW264.7 macrophages [88]. Inhibition of MMP-9 secretion found in this study demonstrated that the macrophageal secretion of a tissue-destructing enzyme is also downregulated, which supports the role of WIN as an anti-inflammatory and tissue-protective drug. Taken together, *in vitro* and *in vivo* studies indicate that the cannabinoid receptor agonist WIN represents a powerful possibility to reduce and limit the activity of the monocyte-macrophage-system, specifically the release of tissue damaging substances such as free oxygen and nitrogen radicals and tissue destroying enzymes.

WIN-induced inhibition of MMP-9-secretion is associated with a strong intracellular accumulation of the 92 kDa mature from of MMP-9, which suggests an inhibitory mechanism in the secretion process. Synthetic inhibitors of MMP-9 act by direct interaction with MMP-9, they include the peptidomimetics batimastat and marimastat, and the non-peptidomimetics tanomastat, prinomastat and BMS-275291 [89]. Other synthetic inhibitors of MMP-9 down-regulate MMP-9-transcription, amongst these tetracycline and its derivatives minocycline, metastat and doxycycline [90], raloxifen [91], nobiletin [92], and rosiglitazone [93]. The intracellular accumulation of MMP-9 in parallel with decreased secretion as shown in our experiments has only been reported in one previous study, in which hypoxia induced a reduction of TNF-α-induced MMP-9 secretion in U937 monocytes [36]. In that study the accumulation was accompanied by decreased MMP-9 in secretory vesicles, and an enhanced surface binding of MMP-9. In contrast to those findings, our study could not detect changes in intracellular distribution of MMP-9 after WIN-treatment with immunfluorescence (fig. 4). Therefore the mechanism of decrease in MMP-9 secretion and intracellular accumulation is likely to be different from the observation in the study mentioned above. Importantly, WIN-induced intracellular accumulation of MMP-9 could result in a strong MMP-9 release after apotosis or necrosis of the macrophages, which should be tested in further long-term *in vitro* or *in vivo* studies.

Post-transcriptional regulation of MMP-9 was described for biphosphonate clodronate [94], statins [95], and native fibronectin [96], which regulate MMP-9 independently of mRNA levels. Among these, an intracellular accumulation of MMP-9 has been ruled out experimentally for fibronectin [96], but it has not been investigated for clodronate and statins.

In our experiments we were able to confirm that MEK/ERK signaling is involved in transcriptional regulation of MMP-9, which has been demonstrated previously in macrophages [35], [83] as well as in other cell types, such as adult rat cardiac fibroblasts [97]. We found surprising evidence that WIN does not use the “classical” cannabinoid receptors to regulate maturation and secretion of MMP-9. Instead we observed that these effects are mimicked by CZP and antagonized by CIC. This points to an antagonistic action of WIN at TRPV1 as a signaling element underlying the WIN-induced effects on MMP-9. TRPV1 plays an important role in immune control: Previous studies using TRPV1-deficient mice revealed protective effects of TRPV1 in mouse models of colonic inflammation [98] and allergic contact dermatitis [99], rendering TRPV1 as a potential pharmacological target for the treatment of inflammatory conditions [100]. Functional antagonism of WIN on TRPV1 has already been shown in several model systems: WIN was found to act as an antagonist on TRPV1 in primary rat trigeminal ganglion cultures, leading to dephosphorylation of the receptor [101] and it evoked antihyperalgesia and antinociception via TRPV in an *in vivo* model for trigenimal and dorsal root ganglia pain [102]. The antagonistic effect of WIN on TRPV1 was also responsible for a reduction in microglia activation in a model for age-associated brain inflammation [78]. However, the protective effects of TRPV1 antagonisation were attributed mainly to neuronal targets rather than to the immune system. Considering the fact that CIC is already active on TRPV1 in low nM ranges as observed in electrophysiology-experimens [103], [104], [105], the concentration of 10 µM used in our experiment is high. However, other effects of TRPV1 on channel activity [106], [107] or on the expression of the inflammatory mediator IL-6 [108] are induced in the µM range. The wide range of functional CIC concentrations in different model systems could be the consequence of different CIC bioavailabilities. Based on our experiments, we cannot rule out that the effects on MMP-9 are also mediated by a CZP/CIC-sensitive element other than TRPV1. We nevertheless suggest that a CIC/CZP-sensitive binding site is involved. The site is probably TRPV1, which transduces anti-inflammatory signals in cells of the monocyte-macrophage-system, which in turn down-regulates MMP-9 maturation and secretion.

Interestingly, the endocannabinoid anandamide also induced apoptosis in human U937 macrophages via TRPV1 [109]. Consequently, TRPV1 cannot only control and limit macrophage activation at a certain threshold, but it also reduces its number and concentration in inflamed tissue. We were able to demonstrate that WIN was capable of inhibiting bone resorption by primary osteoclasts. Since MMP-9 is a crucial element in bone resorption [24] and since the extent of capsaicin-sensitivity of WIN-induced bone protection was comparable to the extent of MMP-9 inhibition (see fig. 11), it is possible that bone protection and MMP-9 inhibition are not only associated, but also causally linked. Modulation of bone homeostasis by agents acting in the cannabinoid system has been reported for CP 55,940, JWH015, AM251 and SR144528 [24], [110], [111]. Furthermore, a regulatory role of TRPV1 in bone homeostasis has been proposed, because capsaicin promoted differentiation of osteoclasts in bone marrow [112]. Moreover, it was suggested that capsazepine inhibits bone resorption in RANKL-generated osteoclastic cultures [113]. Therefore, it is possible that TRPV1 and cannabinoids are playing an important role in the regulation of bone metabolism.

We demonstrated that the cannabinoid receptor agonist WIN reduced MMP-9 secretion *in vivo* and *in vitro*. Because reduction of MMP-9 activity results in less tissue destruction by proteolysis of the ECM, and subsequently in less inflammatory activation and leukocyte recruitment [114], it represents an interesting therapeutic target, not only for inflammatory conditions but also for the inhibition of bone destruction such as during osteoporosis [24]. Because of its anti-inflammatory properties, treatment with WIN has been studied in several models of inflammatory diseases. In a mouse model of arteriosclerosis, the amount of macrophages in plaque lesions was reduced [58], and in a model of PMA-induced inflammation of ear tissue, topical application of WIN decreased leukocyte infiltration [55]. Because MMP-9 promotes migration and invasion of leukocytes [32], [115], [116], [117], the diminished leukocyte infiltration *in vivo* after WIN-treatment could be the consequence of inhibited MMP-9 secretion. Inhibition of MMP-9 by WIN could also initiate neuroprotective effects: Administration of WIN resulted in a reduced macrophageal infiltration of brain tissue and amelioration of neuronal damage in an EAE model [57]. MMP-9 is capable of damaging the blood brain barrier and thereby facilitating the invasion of leukocytes that subsequently lead to demyelination [118], [119]. The proteolytic activity of MMP-9 may also directly induce axon demyelination [120]. Therefore, inhibition of MMP-9 secretion by WIN can be considered as promising strategy of tissue protection during various inflammatory conditions.

Other possible fields of application are inflammatory bowel diseases, where MMP-9 is upregulated in colonic epithelium, leading to the destruction and pathological reorganization of epithelial tissue [47], [121]. Macrophageal secreted MMP-9 is also significantly involved in irreversible tissue destruction and reorganization in periodontal inflammations [122], [123], [124]. In humans, WIN has already been applied therapeutically to decrease the intraocular pressure of human glaucoma resistant to conventional therapies [125]. Systemic application of WIN is limited by its agonistic actions on cannabinoid receptor 1 which lead to psychotropic side effects. Another possibility is the topical application of WIN because of its transdermal permeation [126]. Reduction of tissue destruction during inflammation and parallel avoidance of psychotropic side effects as the consequence of cannabinoid receptor 1 stimulation could also be achieved by the application of other TRPV1-antagonists. TRPV1-antagonists with a proven *in vivo* tolerance are for instance A-425619 (1-Isoquinolin-5-yl-3-(4-trifluoromethyl-benzyl)-urea) [127], or AMG 517 (N-(4-[6-(4-trifluoromethyl-phenyl)-pyrimidin-4-yloxy]-benzothiazol-2-yl)-acetamide I) [128].

We conclude that the control of MMP-9 in the monocyte-macrophage system by a WIN-binding site represents a general and pharmacologically well accessible option for tissue protection during inflammation. Therefore, drugs acting on WIN-binding site may possess the potential to specifically attenuate and limit tissue damage during inflammation, without suppressing the immunological network in general.

## Supporting Information

Figure S1 ß-actin controls of the Western blot in figure 8. The demonstrated Western blot analyses had separate actin controls. In order to arrange the figure 8 for comparison with mRNA quantification, blots were cut and rearranged. ß-actin 1 is from the same blot as p-EKR1/2, ß-actin 2 is from the same blot as MMP-9 and ERK1/2. Western blot analyses of intracellular (MMP-9 cellular) and secreted (MMP-9 secreted) MMP-9 and for phosphorylated (pERK1/2) and unphosphorylated ERK1/2 are demonstrated. U937-macrophages were treated with WIN (4 µM) or the pharmacological ERK1/2 phosphorylation inhibitor U-0126 or PD98059.
(TIF)
